# TRAF4 Inhibits the Apoptosis and Promotes the Proliferation of Breast Cancer Cells by Inhibiting the Ubiquitination of Spindle Assembly-Associated Protein Eg5

**DOI:** 10.3389/fonc.2022.855139

**Published:** 2022-05-25

**Authors:** Miaomiao Hao, Jie Zhang, Mingfang Sun, Kexin Diao, Jian Wang, Shiping Li, Qixue Cao, Shundong Dai, Xiaoyi Mi

**Affiliations:** ^1^ Department of Pathology, College of Basic Medical Sciences, China Medical University, Shenyang, China; ^2^ Department of Pathology, Shanghai Ninth People’s Hospital, Shanghai JiaoTong University School of Medicine, Shanghai, China; ^3^ Department of Pathology, School of Basic Medical Sciences, Hebei University, Baoding, China; ^4^ Department of Pathology, College of Basic Medical Sciences, First Affiliated Hospital, China Medical University, Shenyang, China

**Keywords:** TRAF4, Eg5, breast cancer, mitosis, ubiquitination, Smurf2

## Abstract

Tumor necrosis factor receptor associated factor 4 (TRAF4) is a RING domain E3 ubiquitin ligase that mediates the ubiquitination of various proteins and plays an important role in driving tumor progression. By studying the relationship between TRAF4 and Eg5, a member of the kinesin family that plays a critical role in spindle assembly, we demonstrated that TRAF4 regulated Eg5 ubiquitination and contributed to Eg5-mediated breast cancer proliferation and inhibited breast cancer apoptosis. TRAF4 and Eg5 were both highly expressed in breast cancer and their protein level was positively correlated. Relying on its Zinc fingers domain, TRAF4 interacted with Eg5 in the cytoplasm of breast cancer cells. TRAF4 was a mitosis-related protein, and by up-regulating the protein level of Eg5 TRAF4 participated in spindle assembly. Loss of TRAF4 resulted in monopolar spindles formation, but loss of function could be rescued by Eg5. Relying on its RING domain, TRAF4 up-regulated Eg5 protein levels by inhibition of Eg5 ubiquitination, thus stabilizing Eg5 protein level during mitosis. Furthermore, we found that Smurf2, a TRAF4-targeted ubiquitination substrate, mediated the regulation of Eg5 ubiquitination by TRAF4. TRAF4 inhibited the interaction between Smurf2 and Eg5, and down-regulated the protein level of Smurf2 by promoting its ubiquitination, thereby inhibited the Smurf2-catalyzed ubiquitination of Eg5 and up-regulated Eg5 protein levels. We also demonstrate that TRAF4 plays an important role in promoting cell proliferation and in inhibiting cell apoptosis induced by Eg5. In summary, our study suggests a new direction for investigating the role of TRAF4 in driving breast cancer progression.

## Introduction

The tumor necrosis factor receptor-associated factor (TRAFs) family was originally identified as an adaptor or transducer protein that couples TNF receptors and Toll/interleukin-1 family members to signaling pathways (1, 2). The family members share a C-terminal TRAF homology domain, which mediates their interaction with the TNF receptor super family, thereby activating signal pathways such as NF-κB and MAPK, and participating in the regulation of cell apoptosis, immune response and other life activities (3–6). TRAFs are expressed differently in different human tissues and diseases. Studies have confirmed that TRAF2, TRAF4 and TRAF6 are highly expressed in malignant tumor tissues and play an important role in promoting tumor progression (7, 8).

TRAF4 is a special member of the TRAFs family, since TRAF4 interacts weakly with a few TNF-R family (3–6). TRAF4 protein was discovered in breast cancer-derived metastatic lymph nodes (9). Follow-up studies have found that TRAF4 is overexpressed in a variety of tumor tissues and plays an important role in promoting tumorigenesis and regulating tumor cell proliferation, apoptosis, migration, invasion and other biological behaviors (10–14). It has been reported that TRAF4 can enhance the nuclear protein level of PRMT5 in breast cancer cells, thereby promoting the proliferation of breast cancer cells (15). In endometrial cancer, TRAF4 activates the PI3K/AKT signaling pathway and promotes tumor proliferation and migration (16). Like other family members with the exception of TRAF1, TRAF4 contains a RING domain, which has E3 ubiquitin ligase activity, and participates in regulating the ubiquitination of multiple proteins (17). Ramesh et al. have found that TRAF4 enhances the kinase activity of the NGF receptor TrkA by promoting its ubiquitination, thereby promotes the invasion and metastasis of prostate cancer (18). In addition, it has been reported that TRAF4 participates in regulating tumor microenvironment. TRAF4 promotes the epithelial-mesenchymal transition of non-small cell lung cancer and enhances the proliferation and invasion of tumor cells (19). Xu et al. have found that TRAF4 interacts with many apoptosis-related proteins (20), but the relevant mechanism is still unclear.

To further investigate the putative function of TRAF4, a yeast-two hybrid screen and immunoprecipitation/mass spectrometry were performed by Rozan et al., and several candidate interacting proteins were identified, including mitosis-associated protein Eg5 (21).

The mitotic spindle is a complex organelle consisting of microtubules, motor proteins, nonmotor microtubule-associated proteins, and various other signaling molecules. The formation of a bipolar spindle is crucial for faithful chromosome segregation, ensuring the accurate transmission of genetic information (22). Eg5, a member of the kinesin family, plays a critical role in this process (23, 24). In prophase Eg5 is enriched at spindle poles, while in metaphase and anaphase, Eg5 localizes to antiparallel overlap microtubules in the midzone (25). By cross-linking and sliding of anti-parallel microtubules, Eg5 helps to generate an outward force, which is essential for centrosome separation and bipolar spindle assembly during early mitosis (26–29). Eg5 inhibition results in the formation of monopoles and cells apoptosis (30, 31).

In addition to its role in spindle assembly, Eg5 has been reported to play a role in tumorigenesis (32), and Eg5 is highly expressed in various tumor tissues and cells (33–36). Inhibition of Eg5 suppresses tumor growth (37), whereas over-protein level of Eg5 leads to chromosomal mis-segregation and genomic instability in transgenic mice (23). Therefore, exploring the molecular mechanism of the regulation of Eg5 protein level has important implications.

These results suggest that TRAF4 plays an important role in mitosis spindle assembly by regulating Eg5. TRAF4 up-regulates Eg5 protein levels by inhibiting Eg5 ubiquitination, thus preventing Eg5 degradation (and associated mitotic defects), and in doing so TRAF4 promotes breast cancer cell proliferation and inhibits breast cancer cell apoptosis.

## Materials and Methods

### Patients and Tissue Samples

Breast cancer tissue samples were obtained from patients at the First Affiliated Hospital of China Medical University. Prior informed consent was obtained. All procedures were conducted with the approval of the Ethics Review Committee at the First Affiliated Hospital of China Medical University. All tumor specimens were surgically resected. None of the patients had received chemotherapy or radiotherapy before tumor excision. The pathologic diagnoses were independently identified by three pathologists, based on the World Health Organization guidelines.

### Immunohistochemistry

Surgically removed tumor specimens were fixed in 10% neutral formalin and embedded in paraffin. The specimens were then cut into 4-μm thick sections and baked in an oven at 70°C for 2 h. The tissue sections were dewaxed in xylene, absolute ethanol, gradient alcohol, and distilled water, and then boiled in 0.01M citrate buffer (pH 6.0) at high temperature and pressure for 2min. Endogenous peroxidase activity was blocked with 0.3% hydrogen peroxide, and non-specific binding was blocked with 5% normal goat serum for 30min at 20°C. Tissue sections were then incubated with TRAF4 rabbit polyclonal antibody (H2818, 1:100, Santa Cruz Biotechnology) and Eg5 rabbit polyclonal antibody (ab37814, 1:100, Abcam) at 4°C overnight. Immunochemical reactions were developed using an Elivision super HRP (mouse/rabbit) immunohistochemistry kit (Maixin-Bio, Shenzhen, China) and 3,3-diaminobenzidine (DAB). The nuclei were stained with hematoxylin, and then the sections were dehydrated in ethanol before mounting. TRAF4 and Eg5 protein level levels were evaluated based on the percentage of positive cells (PP) and staining intensity (SI) within the whole tissue section. Staining intensity was evaluated semi-quantitatively using the immune response score (IRS) and calculated using the equation: IRS = PP × SI. PP:0 = no staining; 1 = 1–25%; 2 = 26–50%; 3 = 51–75%; and 4 = 76–100%. SI: 0 = no staining; 1 = weak; 2 = moderate; 3 = strong staining. Each case was then defined as negative (IRS < 3), low protein level (3 = < IRS < 6), or high protein level (6 = < IRS ≤ 12).

### Cell lines and Cell Culture

The non-tumorigenic mammary epithelial cell line MCF-10A, and the breast cancer cell lines MCF-7, MDA-MB-231, SK-BR-3, and MDA-MB-453 were obtained from the Shanghai Cell Bank of the Chinese Academy of Sciences (Shanghai, China) and identified by short tandem repeat (STR) DNA analysis. MCF-10A cells were cultured in 1:1 Dulbecco’s modified Eagle’s medium (DMEM)/F12 (Gibco, Waltham, MA, USA) supplemented with 5% serum, 10μg/mL insulin (Sigma-Aldrich Co, St. Louis, MO, USA), and 20 ng/mL epidermal growth factor (EGF). MCF-7, SK-BR-3 and MDA-MB-453 cells were cultured in DMEM (Gibco, Waltham, MA, USA) supplemented with fetal bovine serum (FBS). MDA-MB-231 cells were cultured in L15 (Gibco, Waltham, MA, USA) supplemented with 10% FBS. All cells were cultured at 37°C in a humidified incubator with 5% CO_2_.

### Plasmid, si-RNA, and Transfection

Plasmids for full-length TRAF4 (wild type, WT) were purchased from MiaolingBio (Wuhan, China), TRAF4 Zinc fingers domain deletion mutant (TRAF4 ΔZn) provided by Dr. Bert W. O’ Malley. Plasmids for TRAF4 RING domain deletion mutant (TRAF4 ΔR), TRAF4 TRAF domain deletion mutant (TRAF4 ΔT) and HA-ubiquitin were purchased from Addgene (Cambridge, USA). Plasmids for Eg5 were purchased from OriGene (Rockville, MD, USA). TRAF4 si-RNA, Smurf2 si-RNA, and Eg5 si-RNA were purchased from RiboBio (Guangzhou, China). Eg5 sh-RNA were purchased from GenePharma (Suzhou, China). Cells were transfected with plasmids using the Attractene Transfection Reagent or with si-RNA using HiPerFect Transfection Reagent (Qiagen, Hilden, Germany) according to the manufacturer’ s protocols. The empty plasmid and scrambled sequences were used as controls.

### Western Blot Analysis

Total protein was extracted from cell lines using lysis buffer (Thermo Fisher Scientific), containing a protease and phosphatase inhibitor cocktail (Beyotime, Shanghai, China), and quantified using the Bradford method. Next, 40 µg of protein was subjected to sodium dodecyl sulfate polyacrylamide gel electrophoresis (8%), and the separated proteins were transferred to polyvinylidene fluoride membranes (EMD Millipore, Billerica, MA, USA). After blocking with 5% non-fat milk in PBS, the membranes were incubated overnight at 4°C with primary antibodies against: TRAF4 (H2818, 1:100, Santa Cruz Biotechnology), Eg5 (1:1000), Smurf2 (F0641, 1:100, Santa Cruz Biotechnology), HA (TA180128S, 1:1000, Origene), α-tubulin (ab52866, 1:1000, Abcam), Caspase-3 (M005851F, 1:1000, Abmart), Bcl-2 (T40056F, 1:1000, Abmart), Bax (T40051F, 1:1000, Abmart), Ki67 (550609, 1:1000, BD Pharmingen), GAPDH (AF0006, 1:2000, Beyotime) and β-actin (1:2000, Beyotime). Next, the membranes were incubated with secondary HRP-conjugated antibody, anti-mouse immunoglobulin G (IgG), or anti-rabbit immunoglobulin (1:2000, Santa Cruz Biotechnology) at 37°C for 2h. Finally, antibody binding wasvisualizedusing electro-chemiluminescence (Thermo Fisher Scientific), and quantified using ImageJ (National Institute of Health, Bethesda, MD, USA).

### Co-Immunoprecipitation

Cell lysates were obtained as described above and precleared by rocking for 2h at 4°C with 20 μL (50% slurry) agarose A/G beads. After the beads were removed, the lysates were incubated with the appropriate antibodies (1–2 μg antibodies per 200 μg protein) at 4°C overnight. Next, 20 μL(50% slurry) agarose A/G beads was added and the samples were rocked for 6 h at 4°C. Finally, the immune complexes were washed with cell lysis buffer and protein bands were detected using immunoblotting assays.

### Immunofluorescence Staining

Breast cancer cells were cultured in 24-well plates for 24 h. The cells were fixed in 4% paraformaldehyde for 15min, and then treated with 0.2% Triton X-100 for 15min to ensure permeabilization. After blocking in 3% BSA for 1h at room temperature, the cells were incubated overnight at 4°C with primary antibodies against: TRAF4 (5100900, 1:100, BD Pharmingen), Eg5 (1:100), and α-tubulin (1:100). Next, the cells were incubated with a tetramethylrhodamine-labeled secondary antibody for 2h at room temperature in the dark. The nuclei were then stained with DAPI. Finally, representative images were captured using an Olympus LH100-3 microscope (Olympus, Tokyo, Japan).

### Cell Proliferation and Colony Formation Assays

Cell viability was measured by the mitochondrial reduction of 3-[4,5-dimethylthiazole-2-yl]-2,5-diphenyltetrazolium bromide (MTT) assay. Cells (3,000 cells/well) were seeded in 96-well plates in medium containing 10% FBS. Samples were treated with MTT solution (10 μL/well) for 4 h. The medium was aspirated from each well and 150μL DMSO was added. The absorbance was then measured at 490nm using a microplate reader. The OD value of a blank was subtracted from the absorbance obtained at each given time. We then calculated the relative ratio, and used these values to plot the cell proliferation curve. For colony formation experiments, cells (1,000/dish) were seeded in 40mm dishes and incubated for 10–15 days. The cells were then fixed with methanol and stained with crystalviolet. The number of colonies with >50 cells was counted.

### Animal Experiments

MCF-7 Cells stably transfected with TRAF4 or co-transfected with TRAF4 and Eg5 sh-RNA were selecting by G418 and Puromycin. Female BALB/c nude mice between five and six weeks of age (Charles River, Beijing, China) were randomly divided into three groups, with five mice in each group and subcutaneously in oculated with 1×10^7^/0.2mL MCF-7, MCF-7/TRAF4 or MCF-7/TRAF4-shEg5 cells. Measured and recorded the tumor volume every 3 days (Tumor volume=1/2 length×width^2^). The animal study was reviewed and approved by the Animal Research Ethics Comittee of China Medical University.

### Statistical Analyses

The Pearson chi-square test and Fisher’s exact test were used to analyze the relationship between TRAF4 and Eg5 protein level. Differences between the groups were analyzed using a paired t-test. Differences with P-values <0.05 were considered statistically significant. All experiments were performed at least three times.

## Results

### The Protein Level of TRAF4 and Eg5 are High in Breast Cancer, and are Positively Correlated

**Figure 1:**
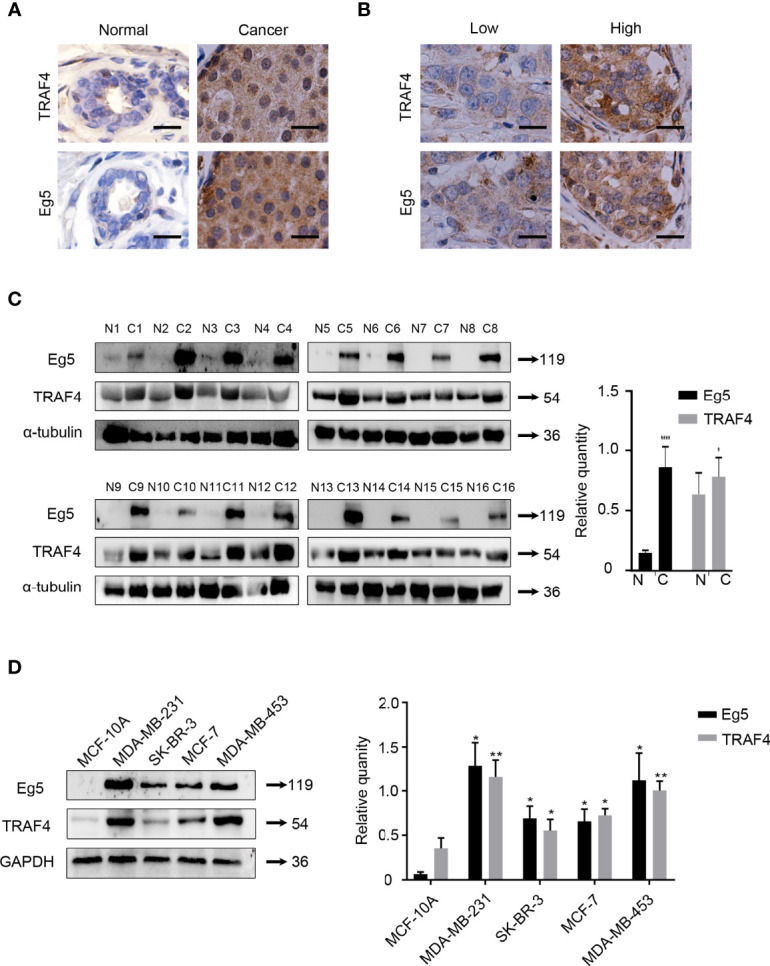
TRAF4 and Eg5 are highly expressed in breast cancer and there is a positive correlation between TRAF4 and Eg5 protein level levels. **(A)** Immunohistochemical analysis demonstrating the protein level and localization of TRAF4 and Eg5 in normal breast tissue (NBT) and breast cancer tissue (BCT). Bar, 20μm. **(B)** The protein level of Eg5 was positively correlated with TRAF4 protein level levels in breast cancer patient tissue. Bar, 20μm. **(C)** Protein level and correlation of TRAF4 and Eg5 in 16 breast cancer patient tissues and paired normal tissues, as detected by western blot. α-tubulin was used as an internal control. ****p < 0.0001. **(D)** protein level of TRAF4 and Eg5 in non-malignant breast epithelial cell line MCF-10A and breast cancer cell lines MDA-MB-231, SK-BR-3, MCF-7 and MDA-MB-453, as analyzed by western blot. GAPDH was used as an internal control. Data represent mean ± SD (*n* = 3). (*p< 0.05, **p<0.01).

To explore the association between TRAF4 and Eg5 in human breast cancer, we compared the protein level of Eg5 and TRAF4 in normal breast tissue (NBT) (n=16) and in breast cancer tissue (BCT) (n=78). As shown in **Table 1**, TRAF4 and Eg5 were either negative or only weakly expressed in normal breast epithelial cells. However, the protein level of both proteins were high in breast cancer cells (**Figure 1A**). We analyzed the association between the protein level of TRAF4 and the Eg5 and found that the protein level of Eg5 and TRAF4 are positively correlated (r=0.7423, p<0.0001; **Figure 1B**; **Table 2**). To confirm these results, we detected the protein level of Eg5 and TRAF4 in matched normal and breast cancer fresh tissues from 16 patients. Consistent with the previous results, the protein level of Eg5 and TRAF4 were high in breast cancer tissues (in comparison with matched normal tissues), and a positive correlation between them was observed (p<0.05, **Figure 1C**). When we compared Eg5 and TRAF4 protein level in a non-malignant breast epithelial cell line (MCF-10A) and in breast cancer cell lines (MDA-MB-231, SK-BR-3, MCF-7and MDA-MB-453), we obtained a similar result (**Figure 1D**). These results indicate that the protein level of TRAF4 and Eg5 are both high in human breast cancer tissues and breast cancer cell lines, and positively correlated, which suggests that TRAF4 and Eg5 may play a promoting role in the occurrence and development of breast cancer.

**Table 1:** The protein level of TRAF4 and Eg5 in tissue.

		TRAF4		Eg5	
	Number	Negative	Positive	Negative	Positive
**Normal Breast Tissue**	16	12	4 (25.00%)	15	1 (6.25%)
**Breast Cancer Tissue**	78	21	57 (73.08%)	17	61 (78.21%)

Fisher’s exact test (TRAF4 p < 0.001; Eg5 p < 0.0001).

**Table 2:** Correlation between TRAF4 and Eg5 protein level in breast cancer tissue.

		TRAF4	
		Negative/Low	High
**Eg5**	**Negative/Low**	31	3
	**High**	5	39

Pearson’s chi-square test (r=0.7423, p < 0.0001).

### TRAF4 Interacts With Eg5 and Up-Regulates Eg5 Protein Level

**Figure 2:**
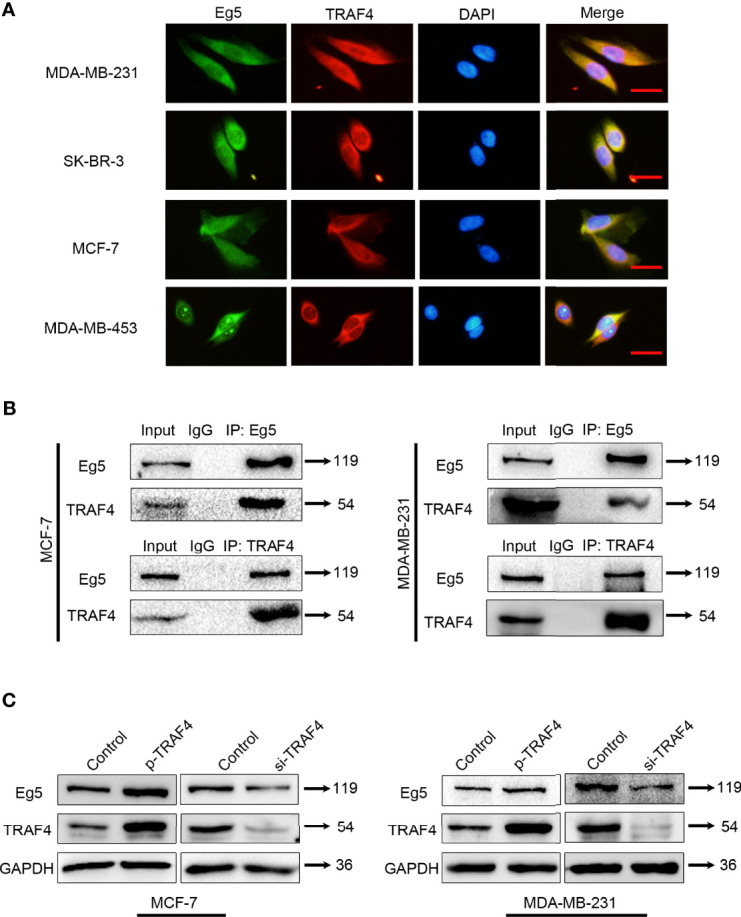
TRAF4 interacts with Eg5 and up-regulates the protein level of Eg5. **(A) **Immunofluorescence assays demonstrated the distribution of endogenous Eg5 and TRAF4 using antibodies against TRAF4 (red) and Eg5 (green). Cell nuclei were stained by DAPI. Bar, 50μm. **(B)** TRAF4 interacted with Eg5. Co-immunoprecipitation assays were performed on MCF-7and MDA-MB-231 cell lysates using antibodies against TRAF4 or Eg5, followed by immunoblotting. **(C)** TRAF4 up-regulated Eg5 protein levels. Proteins were extracted from cells transfected with TRAF4 protein level vector or TRAF4 si-RNA.Western blot assays were used to demonstrate the protein level of TRAF4 and Eg5. GAPDH was used as an internal control.

Eg5 was previously identified as a candidate TRAF4 interacting protein through a yeast-two hybrid screen (22). To confirm that this interaction also occurred in breast cancer cells, we firstly investigated the location of TRAF4 and Eg5 in MDA-MB-231, SK-BR-3, MCF-7 and MDA-MB-453 breast cancer cells, and found both TRAF4 and Eg5 have cytoplasmic localization in these cells (**Figure 2A**). Then we extracted proteins from MCF-7 and MDA-MB-231 cells and performed co-immunoprecipitation using antibodies against TRAF4 or Eg5 followed by immunoblotting. The results demonstrated that TRAF4 co-immunoprecipitated with Eg5 in both MCF-7 and MDA-MB-231 cells (**Figure 2B**). To further investigate the effect of the interaction between TRAF4 and Eg5, we transfected TRAF4 protein level vector into MCF-7 and MDA-MB-231 cells and detected the Eg5 protein level through western blot analysis, the results showed that the protein level of Eg5 was up-regulated. Furthermore, transfection of TRAF4 si-RNA resulted in a reduction in Eg5 protein protein level (**Figure 2C**). The above results demonstrate that TRAF4 and Eg5 interact with each other in the cytoplasm of breast cancer cells. TRAF4 up-regulates the protein level of Eg5, which suggests that Eg5 is a downstream protein of TRAF4, and the cancer-promoting effect of TRAF4 may be achieved by regulating the protein level of Eg5.

### TRAF4 is Involved in Spindle Assembly Through Regulating the Protein Level of Eg5

**Figure 3:**
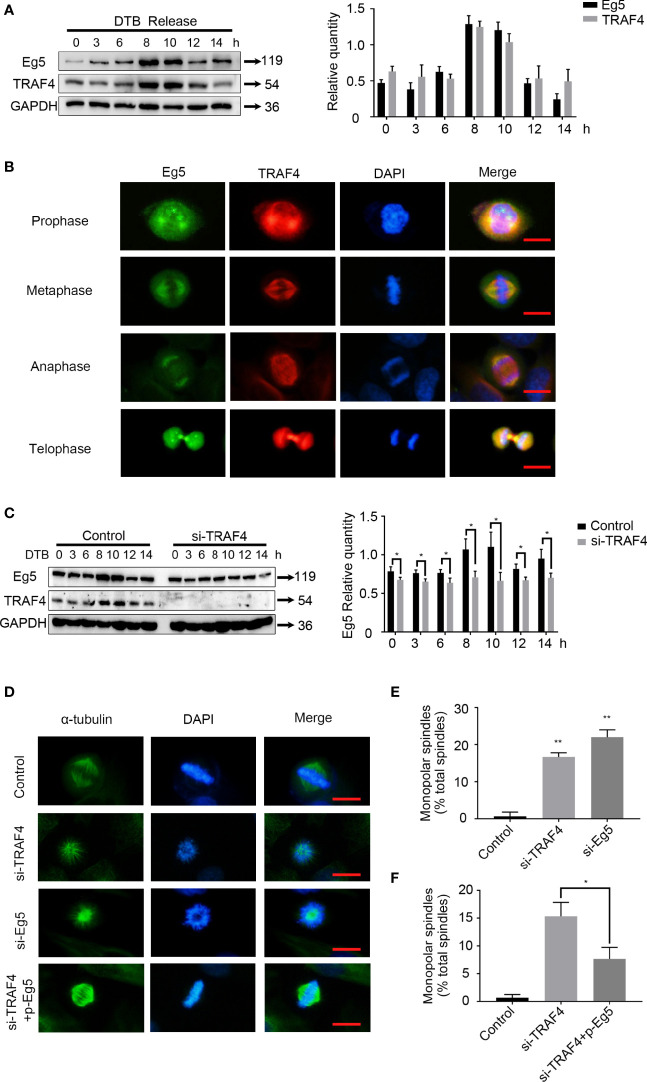
TRAF4 is involved in spindle assembly through regulating the protein level of Eg5. **(A)** MCF-7 cells were synchronized by double thymidine block, and were collected at 0, 3, 6, 8, 10, 12 and 14 hours after release. Western blot assays were used to demonstrate the protein level of TRAF4 and Eg5. GAPDH was used as an internal control. **(B)** Co-localization of endogenous TRAF4 and Eg5 during different phases of mitosis in MCF-7 cells. TRAF4 and Eg5 were detected by immunofluorescence assay using antibodies against TRAF4 (red) and Eg5 (green). Bar, 20μm. **(C)** MCF-7 cells were synchronized by double thymidine block and transfected with or without TRAF4 si-RNA. Collected the cells at the time mentioned above and protein level were analyzed by western blot. Data represent mean ± SD (*n* = 3). (*p< 0.05). **(D)** Depletion of TRAF4 and Eg5 led to monopolar spindle formation. Synchronized MCF-7 cells were transfected with the indicated si-RNAs. Immunofluorescence assays were used to evaluate spindle morphology using antibodies against α-tubulin. Chromosomes were stained by DAPI. Bar, 20μm. **(E)** Summary of the frequency of monopolar spindles in MCF-7 cells transfected with control si-RNA, TRAF4 si-RNA or Eg5 si-RNA. **p < 0.001. **(F)** Summary of the frequency of monopolar spindle in MCF-7 cells transfected with control si-RNA or TRAF4 si-RNA with or without Eg5 protein level vector. Data represent mean ± SD (*n* = 3). (*p< 0.05).

Since Eg5 is a mitotic associated protein, we investigated whether TRAF4 also plays a role during mitosis. We detected the protein level of TRAF4 and Eg5 in MCF-7 cells released from double thymidine block (DTB) for different periods of time. As observed with Eg5 (**Figure 3A**), the protein level levels of TRAF4 exhibited a slight increase 8–10 h after DTB release, suggesting that TRAF4 may be a cell cycle regulated protein. We further investigated the location of these two proteins in mitotic MCF-7 cells, and found that TRAF4 was localized with Eg5 throughout every stage of mitosis (**Figure 3B**).

We then detectected the protein level of Eg5 in thymidine synchronized MCF-7 cells which were transfected with or without TRAF4 si-RNA. The results showed that the protein level of Eg5 during 8-10h after DTB release was significantly reduced in TRAF4-depleted cells comparing with in control cells (**Figure 3C**). These data indicate that TRAF4 mainly positively regulates the protein level of Eg5 protein during mitotic phase, and may be involved in the regulation of cell mitosis by up-regulating the protein level of Eg5.

As previously stated, Eg5 drives bipolar spindle assembly by participating in centrosome separation, and inhibition of Eg5 causes monopolar spindle formation (23, 24). To confirm this result, we transfected Eg5 si-RNA into MCF-7 cells (**Figure S1**) and performed immunofluorescent staining using an antibody against α-tubulin to examine the mitotic spindle. We observed a higher frequency of monopolar spindle formation (**Figures 3D, E**). Since we have verified that TRAF4 regulated the protein level of Eg5 protein during mitotic phase, we hypothesized that TRAF4 plays a role in spindle assembly. We transfected TRAF4 si-RNA into MCF-7 cells and performed immunofluorescent staining using an antibody against α-tubulin to examine the mitotic spindle. We observed a higher frequency of single spindle formation, which could be partially rescued by co-transfection ofthe Eg5 protein level vector (**Figures 3D–F**). These results provide evidence that TRAF4 participates in spindle assembly through regulating the protein level of Eg5.

### TRAF4 Up-Regulates Eg5 by Inhibiting Eg5 Ubiquitination

**Figure 4:**
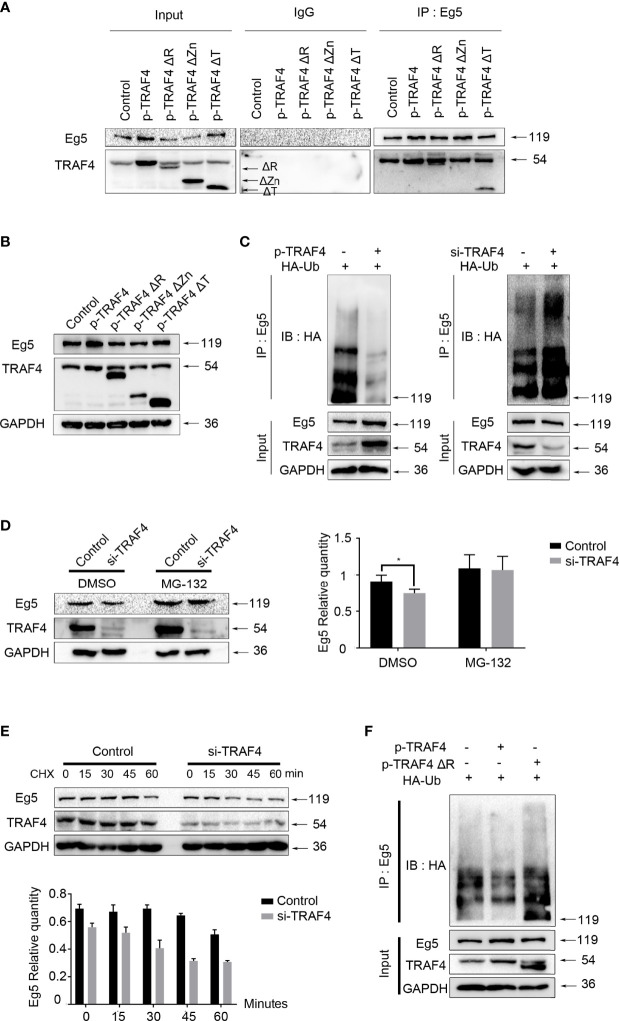
TRAF4 up-regulates Eg5 by inhibiting Eg5 ubiquitination. **(A)** MCF-7 cells were transfected with wild type TRAF4, TRAF4ΔR, TRAF4ΔZn or TRAF4ΔT. Co-immunoprecipitation assays were performed using antibodies against Eg5, followed by immunoblotting. **(B)** Proteins were extracted from MCF-7 cells transfected with wild type TRAF4, TRAF4ΔR, TRAF4ΔZn or TRAF4ΔT. Western blot assays were used to demonstrate the protein level of TRAF4 and Eg5. GAPDH was used as an internal control. **(C)** TRAF4 inhibited the ubiquitination of Eg5. MCF-7cells were co-transfected as indicated. Co-immunoprecipitation was carried out to detected the ubiquitination levels of Eg5, followed by western blot analysis using an antibody against HA. **(D)** TRAF4 protected Eg5 from proteasome-dependent pathway degradation. MCF-7 cells were transfected with TRAF4 si-RNA treated with DMSO or MG132. Western blot assays were performed to evaluate changes in the protein level of TRAF4, Eg5 and GAPDH. Data represent mean ± SD (*n* = 3). (*p< 0.05). **(E)** MCF-7 cells synchronized by thymidine were transfected with Negative control si-RNA or TRAF4 si-RNA, and released in the G2/M phase. Added Cycloheximide at a final concentration of 50mg/ml, and collected cells after 0, 15, 30, 45 and 60 minutes respectively. Western blot assays were performed to evaluate changes in the protein level of TRAF4, Eg5 and GAPDH. **(F)** Regulation of Eg5 ubiquitination level by TRAF4 was dependent on its RING domain. MCF-7cells were co-transfected with HA-ubiquitin and empty vector or TRAF4 vector or TRAF4 RING domain deletion mutant vector. Co-immunoprecipitation was carried out to detected the ubiquitination levels of Eg5, followed by western blot analysis using an antibody against HA.

Next, we investigated the mechanism by which TRAF4 regulating Eg5. To identify the potential interaction domain of TRAF4 and Eg5, we transfected wild type TRAF4, RING domain deleted (TRAF4ΔR); Zinc fingers domain deleted (TRAF4ΔZn) or TRAF domain deleted (TRAF4ΔT) mutants into MCF7, and carried out co-immunoprecipitation experiment. As shown in **Figure 4A**, TRAF4 with RING domain deletion mutant and TRAF4 with TRAF domain deletion mutant could still interact with Eg5, while TRAF4 with Zinc fingers domain deletion mutant could not be dragged down by Eg5 antibody. These results suggested that Zinc fingers domain of TRAF4 was essential for the interaction between TRAF4 and Eg5.

To confirm which domain of TRAF4 was responsible for up-regulating Eg5, we transfected wild type TRAF4 or domain-deleted mutants into MCF-7, and detected the protein level of Eg5 by western blot. The results showed that either over-expressing wild type TRAF4 or TRAF4ΔT could up-regulated Eg5 protein, while TRAF4ΔZn could not regulate Eg5 protein protein level levels, and to our surprise, RING domain deleted also resulted in TRAF4 losing the function of up-regulating the protein level of Eg5 (**Figure 4B**).

Since RING domain of TRAF4 has E3 ligase activity and is involved in the regulation of Eg5 protein level. We determined whether TRAF4 regulated Eg5 through affecting its ubiquitination. We transfected HA-ubiquitin and TRAF4 protein level vectors or TRAF4 si-RNA into MCF-7 cells and performed a ubiquitination assay. Eg5 ubiquitination levels were then detected by co-immunoprecipitation using an antibody against Eg5, followed by western blot analysis using antibodies against HA. Compared with the control group, over-protein level of TRAF4 reduced Eg5 ubiquitination. Moreover, inhibition of TRAF4 enhanced the ubiquitination of Eg5 (**Figure 4C**). These data indicated that TRAF4 inhibited the ubiquitination of Eg5. To confirm this result, we transfected TRAF4 specific si-RNA into MCF-7 cells treated with DMSO or MG-132, and detected Eg5 protein levels through western blot analysis. We found that when the proteasome-dependent pathway was blocked, the down-regulation of Eg5 caused by TRAF4 knockdown was greatly weakened (**Figure 4D**).

In order to explore the significance of the inhibition of Eg5 ubiquitination by TRAF4 during mitosis, we transfected TRAF4 si-RNA into thymidine synchronized MCF-7 cells, and released them in the G2/M phase. Then we carried out cycloheximide protein tracking experiments to detected the half-life of Eg5 during mitosis. The result showed that the Eg5 protein started to decrease at 60 minutes in the control group, while after depleting TRAF4 the time was advanced to 30 minutes (**Figure 4E**). These results indicate that depleting TRAF4 leads to a reduction of Eg5 half-life during mitosis, suggesting that TRAF4 improve the stability of Eg5 protein during mitosis through inhibiting the ubiquitination of Eg5.

Furthermore, we transfected HA-ubiquitin and TRAF4 or TRAF4ΔR into MCF-7 cells and performed a ubiquitination assay to investigate whether TRAF4 relies on its RING domain to regulate Eg5 ubiquitination. The result revealed that only wild-type TRAF4, not TRAF4ΔR, could reduce Eg5 ubiquitination (**Figure 4F**).

### Smurf2 Mediates TRAF4 Inhibition of Eg5 Ubiquitination

**Figure 5:**
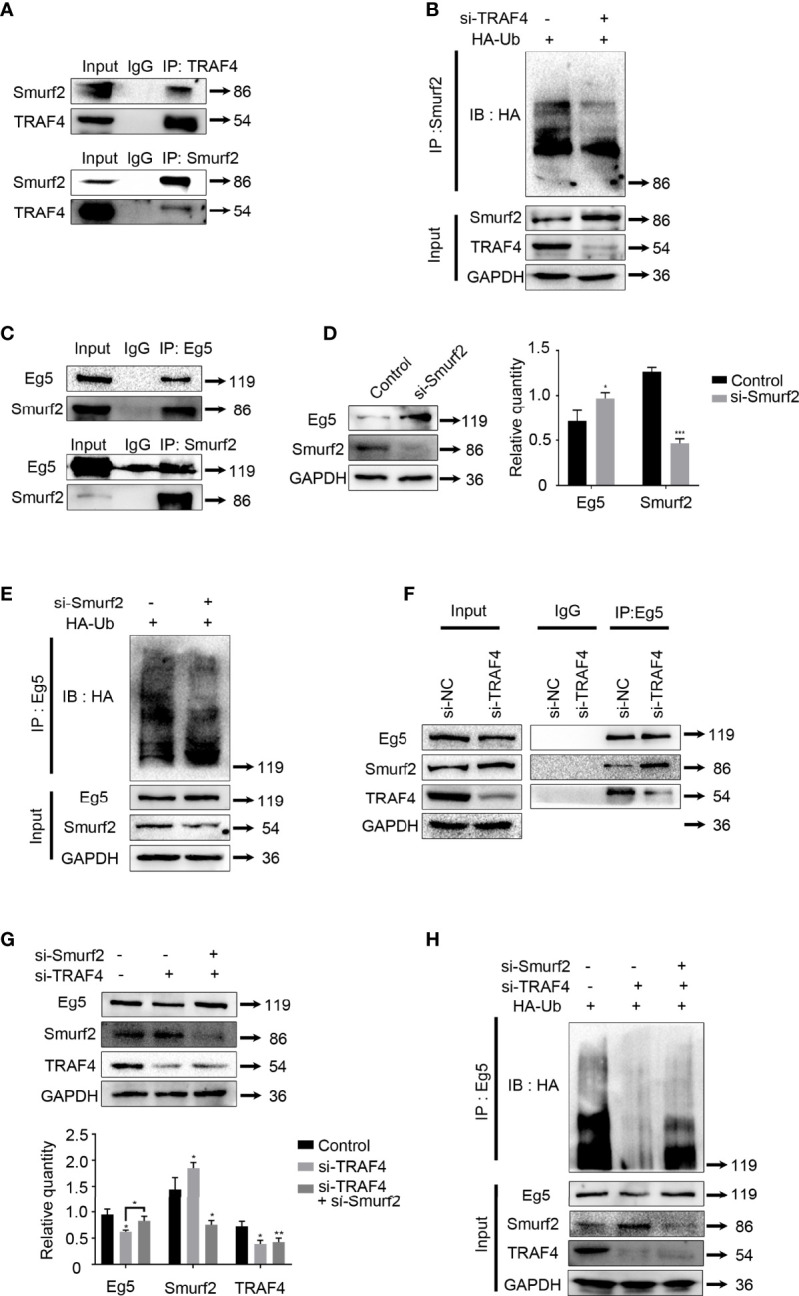
Smurf2 mediates TRAF4 inhibition of Eg5 ubiquitination. **(A)** TRAF4 interacts with Smurf2. Co- immunoprecipitation assays were performed on MCF-7 cells lysates using antibodies against TRAF4 or Smurf2, followed by western blot analysis. **(B)** TRAF4 promoted the ubiquitination of Smurf2. MCF-7cells were co-transfected with HA-ubiquitin and TRF4 si-RNA. Co-immunoprecipitation was carried out to detected the ubiquitination levels of Smurf2, followed by western blot analysis using an antibody against HA. **(C)** Smurf2 interacted with Eg5. Co- immunoprecipitation assays were performed on MCF-7 cells lysates using antibodies against Eg5 or Smurf2, followed by western blot analysis. **(D)** Smurf2 inhibited the protein level of Eg5. The protein level levels of Smurf2, Eg5 and GAPDH in MCF-7cells transfected with Smurf2 si-RNA were evaluated by western blot analysis. Data represent mean ± SD (*n* = 3). (*p< 0.05, ***p<0.001). **(E)** Smurf2 promoted the ubiquitination of Eg5. MCF-7cells were co-transfected with HA-ubiquitin and Smurf2 si-RNA. Co-immunoprecipitation was carried out to detected the ubiquitination levels of Eg5, followed by western blot analysis using an antibody against HA. **(F)** TRAF4 inhibits the interaction between Smurf2 and Eg5. MCF-7 cells were transfected with or TRAF4si-RNA. Co- immunoprecipitation assays were performed using antibodies against Eg5, followed by western blot analysis. **(G)** TRAF4 regulated the protein level of Eg5 protein level through Smurf2. MCF-7 cells were transfected with control si-RNA or Traf4 si-RNA with or without Smurf2 si-RNA. The protein level levels of Smurf2, Eg5 and GAPDH were analyzed by western blot analysis. Data represent mean ± SD (*n* = 3). (*p< 0.05, **p<0.01). **(H)** Smurf2 mediates TRAF4 inhibition of the ubiquitination of Eg5. MCF-7 cells were co-transfected with HA-ubiquitin protein level vector and TRAF4 si-RNA with or without Smurf2 si-RNA. Eg5 ubiquitination levels were detected following co-immunoprecipitation using antibodies against Eg5 by western blot analysis using an antibody against HA.

According to the biogrid database, SMAD-specific E3 ubiquitin protein ligase family member1 (Smurf1) is associated with both TRAF4 and Eg5 (38, 39). However, Smurf1 protein level was constant throughout the cell cycle, while Smurf2, another SMAD-specific E3 ubiquitin protein ligase family member, was highly expressed during mitosis phase in HeLa cells (40). In addition, Smurf2 is also localized at centrosomes (40), and this is coincident with the localization of TRAF4 and Eg5 during the prophase. Thus, we determined whether Smurf2 participates in the regulation of Eg5 by TRAF4. Smurf2 has previously been reported as a substrate of TRAF4 (41).To confirm these results, we performed co-immunoprecipitation and ubiquitination assay in MCF-7 cells and verified that TRAF4 interacted with Smurf2, and down-regulation of TRAF4 leaded to a decrease in the ubiquitination level of Smurf2 (**Figures 5A, B** and **Figure S3**). In addition, we detected the protein level of Smurf2 in a non-malignant breast epithelial cell line (MCF-10A) and in breast cancer cell lines (MDA-MB-231, SK-BR-3, MCF-7and MDA-MB-453), as shown in **Figure S2**.

Then we carried out co-immunoprecipitation using antibodies against Smurf2 or Eg5, followed by immunoblotting. We observed that Smurf2 and Eg5 interacted with each other in MCF-7 cells (**Figure 5C**). To further investigate whether Smurf2 regulates the protein level of Eg5 protein, we transfected Smurf2 si-RNA, and detected the protein level of Eg5 protein by western blot. The results showed that transfection of Smurf2 si-RNA resulted in an increase in Eg5 protein protein level (**Figure 5D**).

Since Smurf2 is an E3 ubiquitin ligase (42), we examined Eg5 ubiquitination levels in cells co-transfected with HA-ubiquitin and Smurf2 si-RNA, and found a reduction of the polyubiquitinated forms of Eg5 (**Figure 5E**). The results indicate that Smurf2 down-regulated Eg5 by promoting its ubiquitination.

Based on the above results, we determined whether TRAF4 up-regulated Eg5 by inhibiting the Smurf2-catalyzed ubiquitination of Eg5, thereby up-regulating the protein level of Eg5. We first tested whether the presence of TRAF4 could affect the interacting between Smurf2 and Eg5. We carried out co-immunoprecipitation and found that after TRAF4 was depleted, the interacting between Smurf2 and Eg5 increased significantly. The result indicates that TRAF4 inhibits the interaction between Smurf2 and Eg5 (**Figure 5F**). To further explore the relationship between TRAF4, Smurf2 and Eg5, we transfected control si-RNA or TRAF4 si-RNA with or without Smurf2 si-RNA into MCF-7 cells, and found that knockdown of Smurf2 reduced Eg5 down-regulation caused by TRAF4 depletion (**Figure 5G**). Next, we examined whether Smurf2 plays a role in the regulation of Eg5 ubiquitination mediated by TRAF4. We transfected HA-ubiquitin protein level vector and TRAF4 si-RNA with or without Smurf2 si-RNA into MCF-7 cells, and performed co-immunoprecipitation using antibodies against Eg5, followed by western blot analysis using antibodies against HA to detect Eg5 ubiquitination. Smurf2 depletion markedly reduced the up-regulation of Eg5 ubiquitination caused by TRAF4 knockdown (**Figure 5H**).

These results suggest that TRAF4 regulates the ubiquitination of Eg5 in a smurf2 dependent manner. TRAF4 inhibits the interaction between Smurf2 and Eg5, and down-regulates the protein level of Smurf2 by promoting its ubiquitination, thereby inhibits the Smurf2-catalyzed ubiquitination of Eg5 and up-regulates Eg5 protein levels.

### TRAF4 Inhibits the Apoptosis and Promotes the Proliferation of Breast Cancer Cells

**Figure 6:**
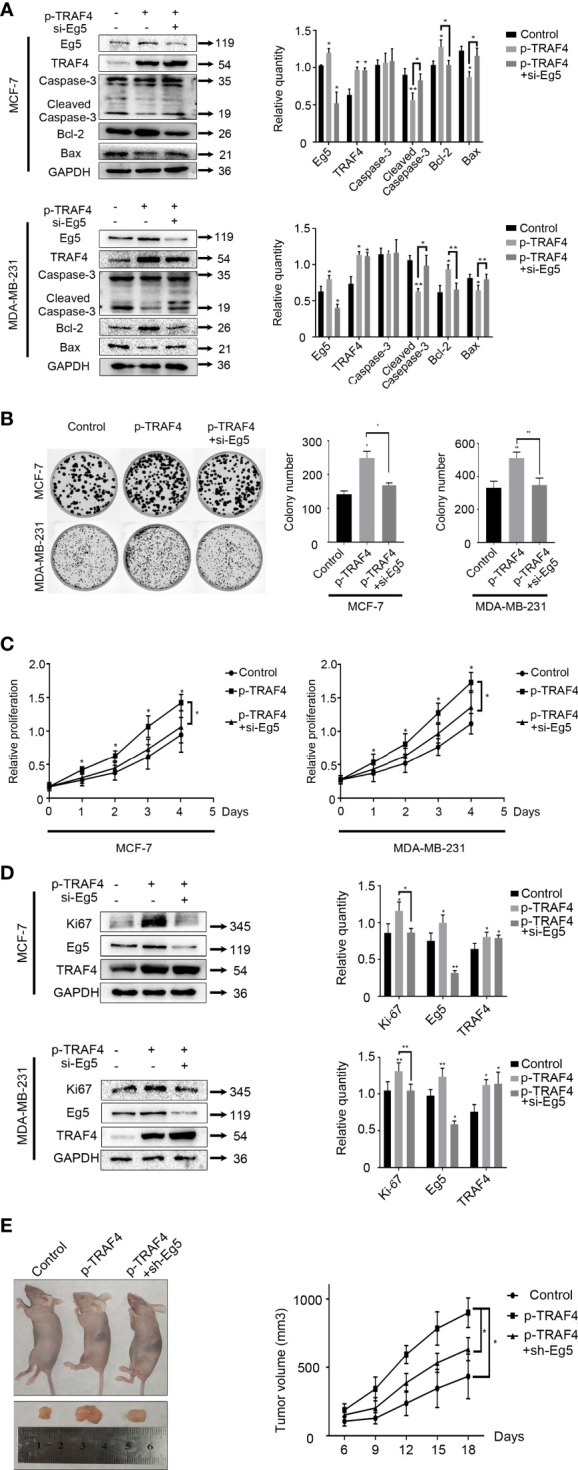
TRAF4 inhibits the apoptosis and promotes the proliferation of breast cancer cells. **(A)** TRAF4 inhibits the apoptosis of breast cancer cells. MCF-7 cells and MDA-MB-231 cells were transfected with TRAF4 si-RNA or co-transfected with TRAF4 si-RNA and Eg5 over-protein level plasmid. Western blot assays were used to demonstrate the protein level of apoptosis-related protein. GAPDH was used as an internal control. Data represent mean ± SD (*n* = 3). (*p< 0.05, **p<0.01). **(B)** TRAF4 promotes the proliferation of breast cancer cells. MCF-7 cells and MDA-MB-231 cells were transfected as indicated. Representative images and quantification of colony formation assays. Data represent mean ± SD (*n* = 3). (*p< 0.05, **p<0.01). **(C)** MCF-7 cells and MDA-MB-231 cells were co-transfected as indicated. An MTT assay was performed to assessproliferation activity in MCF-7 cells and MDA-MB-231 cells. Data represent mean ± SD (*n* = 3). (*p< 0.05). **(D)** MCF-7 cells and MDA-MB-231 cells were transfected as indicated. Western blot assays were used to demonstrate the protein level of Ki-67, Eg5 and TRAF4. GAPDH was used as an internal control. Data represent mean ± SD (*n* = 3). (*p< 0.05, **p<0.01). **(E)** MCF-7 cells transfected as indicated were transplanted into female athymic nude mice. Tumors size was measured every three days. Data represent mean ± SD (*n* = 4). (*p< 0.05).

We have previously confirmed that TRAF4 plays an important role in the assembly process of the mitotic spindle by up-regulating the protein level of Eg5. Next, we will further study how TRAF4 and Eg5 regulate the biological behavior of tumor cells. We transfected TRAF4 plasmid into MCF-7 and MDA-MB-231 cells, and detected the protein level of apoptosis-related proteins Caspase-3, Cleaved Caspase-3, anti-apoptotic protein Bcl-2 and pro-apoptotic protein Bax. The results showed that over-protein level of TRAF4 caused the protein level of Cleaved Caspase-3 and Bax decrease, while the protein level of Bcl-2 increase. Then we co-transfected TRAF4 plasmid and Eg5 si-RNAs and carried out western blot, and found that the effect on these three proteins caused by over-protein level of TRAF4 was weakened (**Figure 6A**). The above results indicate that TRAF4 inhibits breast cancer cells apoptosis by up-regulating the protein level of Eg5 protein.

Then we investigated the role of TRAF4 and Eg5 in cell proliferation through cell viability assay and colony formation assays. The results showed that TRAF4 over-protein level promoted MCF-7 and MDA-MB-231 cells proliferation, while co-transfecting TRAF4 plasmid and Eg5 si-RNA significantly reduced the TRAF4-induced proliferation of breast cancer cells (**Figures 6B, C**). Similarly, we detected an increased protein level of ki-67 in MCF-7 and MDA-MB-231 cells after transfecting with TRAF4 plasmid, and the increased was weakened when we co-transfected TRAF4 plasmid and Eg5 si-RNA (**Figure 6D**). These results suggest that TRAF4 promotes the proliferation of breast cancer cells by up-regulating Eg5 protein levels. In order to confirm the role of TRAF4 and Eg5 in the development of breast cancer *in vivo*, we transplanted breast tumours developed from MCF-7 cells, which were stably transfected with empty plasmid or TRAF4 overexpressed pasmid or both TRAF4 overexpressed pasmid and Eg5 sh-RNAs into nude mice. We measured the tumors size every three days and draw growth curves. The results showed that the growth of tumors over-expressing TRAF4 was significantly accelerated, and when Eg5 was depleted at the same time, the promotion effect of TRAF4 on tumor growth was inhibited (**Figure 6E**), verifying that TRAF4 promotes the proliferation of breast cancer cells by up-regulating the protein level of Eg5.

## Discussion

TRAF4, a protein originally discovered in breast cancer-derived metastatic lymph nodes (9), has been shown to play an important role in breast cancer progression (15, 41). Previous studies have confirmed that TRAF4 promoting tumor proliferation, migration, invasion through various pathways (10–14), but the mechanism by which TRAF4 inhibits apoptosis is not yet clear. In the present study, we found that Eg5, which has the function of inhibiting apoptosis, and TRAF4 are both highly expressed in breast cancer, and that the protein level of TRAF4 and Eg5 are positively correlated. We demonstrate that TRAF4 and Eg5 interact with each other, and TRAF4 has a positive regulatory effect on the protein level of Eg5 protein.

Since Eg5 is a mitosis-associated protein, which plays a critical role in bipolar spindle assembly in prophase (4–6), we speculate whether TRAF4 participates in mitosis by up-regulating the protein level of Eg5. We demonstrate that the protein level of TRAF4 during mitosis is higher than that during interphase, suggesting that TRAF4 may be involved in the regulation of cell mitosis. We further find that TRAF4 localizes with Eg5 throughout every stage of mitosis and positively regulates the protein level of Eg5 protein during mitotic phase. Moreover inhibition of TRAF4 results in the formation of monopoles, and this defects could be rescued by Eg5. These results suggest that TRAF4 plays an important role in mitosis by regulating Eg5-media spindle assembly in prophase.

We further explored the molecular mechanism of TRAF4 regulating Eg5. TRAF4 contains three functional domains, including a C-terminal TRAF homology domain, a N-terminal RING domain, and seven Zinc fingers motifs domain. The TRAF domain mainly mediates the interaction between TRAF4 and the members of TNF-R family (3–6); the RING domain has E3 ligase activity and participates in regulating protein ubiquitination (17); the Zinc fingers domain mediates the interaction of TRAF4 with other proteins (43). We confirmed that TRAF4 interacts with Eg5 depending on its Zinc fingers domain. Interestingly, we find TRAF4 up-regulates Eg5 depending not only on Zinc fingers domain but also on RING domain. We further research and reveal that TRAF4 up-regulates Eg5 protein levels by inhibiting Eg5 ubiquitination, and confirm that depleting TRAF4 leads to a reduction of Eg5 half-life during mitosis. These results suggest that TRAF4 improve the stability of Eg5 protein during mitosis through inhibiting the ubiquitination of Eg5.

TRAF4 has E3 ubiquitin ligase activity, but has a negative regulatory effect on the ubiquitination of Eg5. We therefore speculate that the regulation of Eg5 ubiquitination by TRAF4 may be mediated by other proteins. We checked databases and literature and found that Eg5 may interact with Smurf2. Smurf2 is a HECT E3 ubiquitin ligase. As mentioned above, Smurf2 is a known ubiquitination substrate of TRAF4, and it has been shown to mediate TRAF4-induced TGF-β receptor signaling (41). In addition, Smurf2 is a regulator of mitosis. Together with the NEDD9 complex, Smurf2 activates Aurrora A, promoting timely cell entry into mitosis (44). Smurf2 is also required for the spindle assembly checkpoint, mediating Mad2 stabilization (40). In comparison with its role during the S phase and the prometaphase, the function of Smurf2 during prophase is less well understood.

Our study confirm that Smurf2 interacts with Eg5, and down-regulates the protein level of Eg5 by promoting its ubiquitination. We further explore the relationship between TRAF4, Smurf2 and Eg5, and find TRAF4 inhibits the interaction between Smurf2 and Eg5. In addition, inhibition of Eg5 ubiquitination by TRAF4 knockdown is weakened when Smurf2 is depleted. These results suggest that TRAF4 inhibites the interaction between Smurf2 and Eg5, and down-regulates the protein level of Smurf2 by promoting its ubiquitination, thereby inhibites the Smurf2-catalyzed ubiquitination of Eg5 and up-regulated Eg5 protein levels.

Finally, we confirm that through up-regulating the protein level of Eg5, TRAF4 down-regulates the protein level of Cleaved Caspase-3, pro-apoptotic protein Bax, and up-regulates the anti-apoptotic protein Bcl-2, indicating that TRAF4 inhibits breast cancer cell apoptosis by up-regulating the protein level of Eg5 protein. Through cell viability assay, colony formation assays and nude mice tumorigenesis experiment, we demonstrate TRAF4 promotes the proliferation of breast cancer cells by up-regulating Eg5 protein levels. Our study uncovers a new aspect of TRAF4 function in spindle assembly through regulation of Eg5 protein levels during mitosis, providing a new research direction for elucidating the role of TRAF4 in driving breast cancer progression.

## Data Availability Statement

The original contributions presented in the study are included in the article/**Supplementary Material**. Further inquiries can be directed to the corresponding author.

## Ethics Statement

The animal study was reviewed and approved by Animal Research Ethics Committee of China Medical University. Written informed consent was obtained from the individual(s) for the publication of any potentially identifiable images or data included in this article.

## Author Contributions

XM, SD, JZ, MS, KD and MH contributed conception and design of the study. MH, JZ, MS, KD, JW, SL and QC performed experiment and the statistical analysis. MH and MS wrote the first draft of the manuscript. All authors contributed to manuscript revision, read, and approved the submitted version.

## Funding

This work was supported by the National Natural ScienceFoundation of China (grant no. 81572615 to XM).

## Conflict of Interest

The authors declare that the research was conducted in the absence of any commercial or financial relationships that could be construed as a potential conflict of interest.

## Publisher’s Note

All claims expressed in this article are solely those of the authors and do not necessarily represent those of their affiliated organizations, or those of the publisher, the editors and the reviewers. Any product that may be evaluated in this article, or claim that may be made by its manufacturer, is not guaranteed or endorsed by the publisher.

## Supplementary Material

Supplementary Figure 1 Protein level of Eg5. Proteins were extracted from cells transfected with Eg5 si-RNA.Western blot assays were used to demonstrate the protein level of Eg5. GAPDH was used as an internal control. Data represent mean ± SD (*n* = 3). (***p<0.001).

Supplementary Figure 2 Protein level of TRAF4, Eg5 and Smurf2 in non-malignant breast epithelial cell line MCF-10A and breast cancer cell lines MDA-MB-231, SK-BR-3, MCF-7 and MDA-MB-453, as analyzed by western blot. GAPDH was used as an internal control. Data represent mean ± SD (*n* = 3). (**p*< 0.05, **p<0.01, ***p<0.001).

Supplementary Figure 3 Co-immunoprecipitation assays were performed on MCF-7 cell lysates using antibodies against TRAF4, Eg5 or Smurf2, followed by immunoblotting.
