# Supplementary figures and images for: TRAF4 Inhibits the Apoptosis and Promotes the Proliferation of Breast Cancer Cells by Inhibiting the Ubiquitination of Spindle Assembly-Associated Protein Eg5
Source: Front Oncol. 2022 May 25;12:855139. doi: 10.3389/fonc.2022.855139 (PMC9174544; doi:10.3389/fonc.2022.855139)

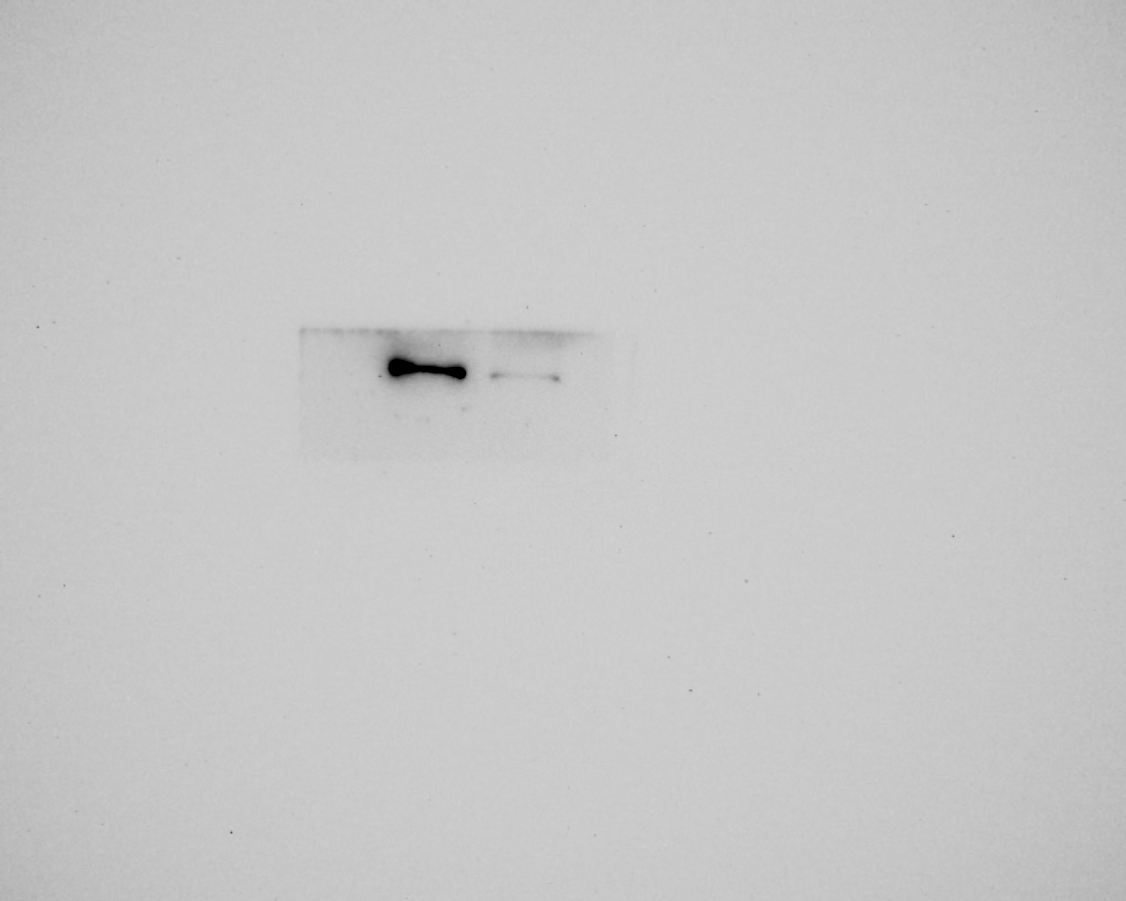

Supplement: Supplementary Figure 1 — Protein level of Eg5. Proteins were extracted from cells transfected with Eg5 si-RNA.Western blot assays were used to demonstrate the protein level of Eg5. GAPDH was used as an internal control. Data represent mean ± SD (n = 3). (***p<0.001). [file DataSheet_1.zip › supplementary results/S1/S1 Eg5.tif]

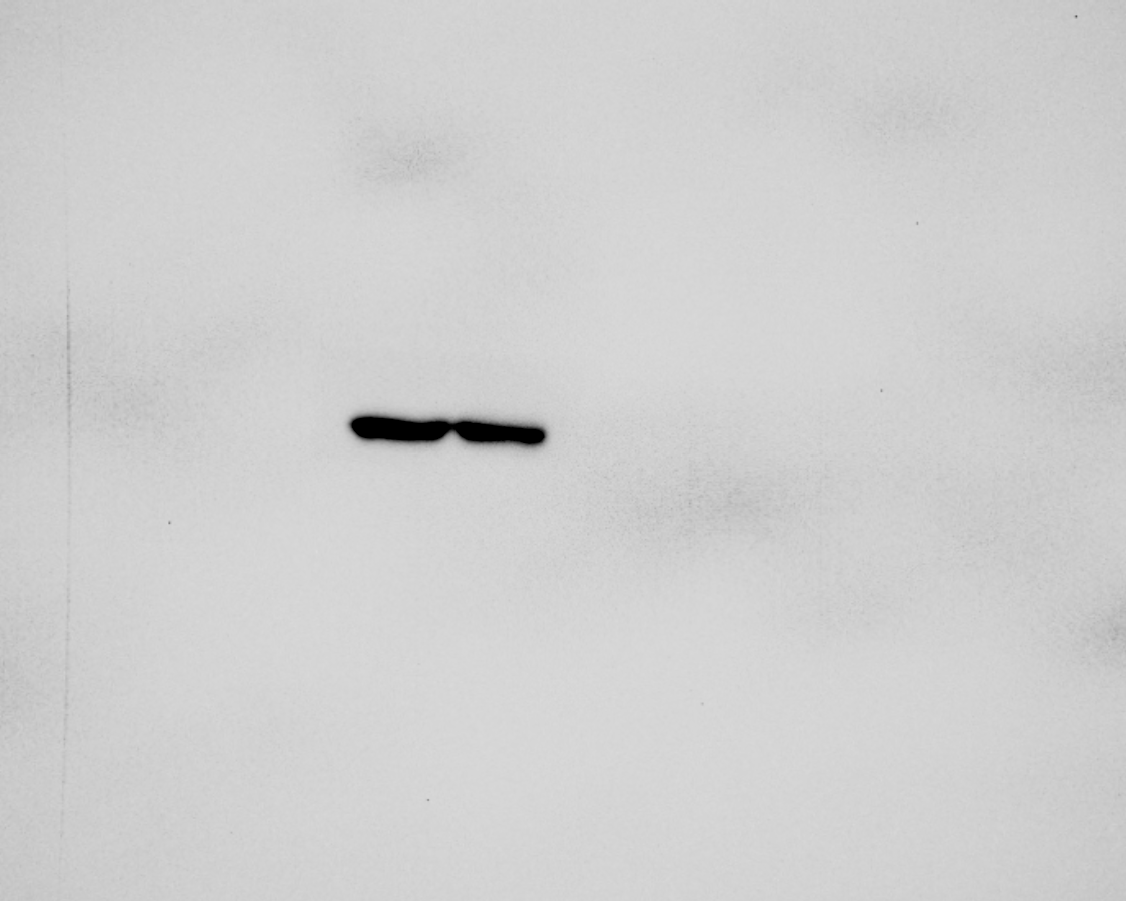

Supplement: Supplementary Figure 1 — Protein level of Eg5. Proteins were extracted from cells transfected with Eg5 si-RNA.Western blot assays were used to demonstrate the protein level of Eg5. GAPDH was used as an internal control. Data represent mean ± SD (n = 3). (***p<0.001). [file DataSheet_1.zip › supplementary results/S1/S1 GAPDH.tif]

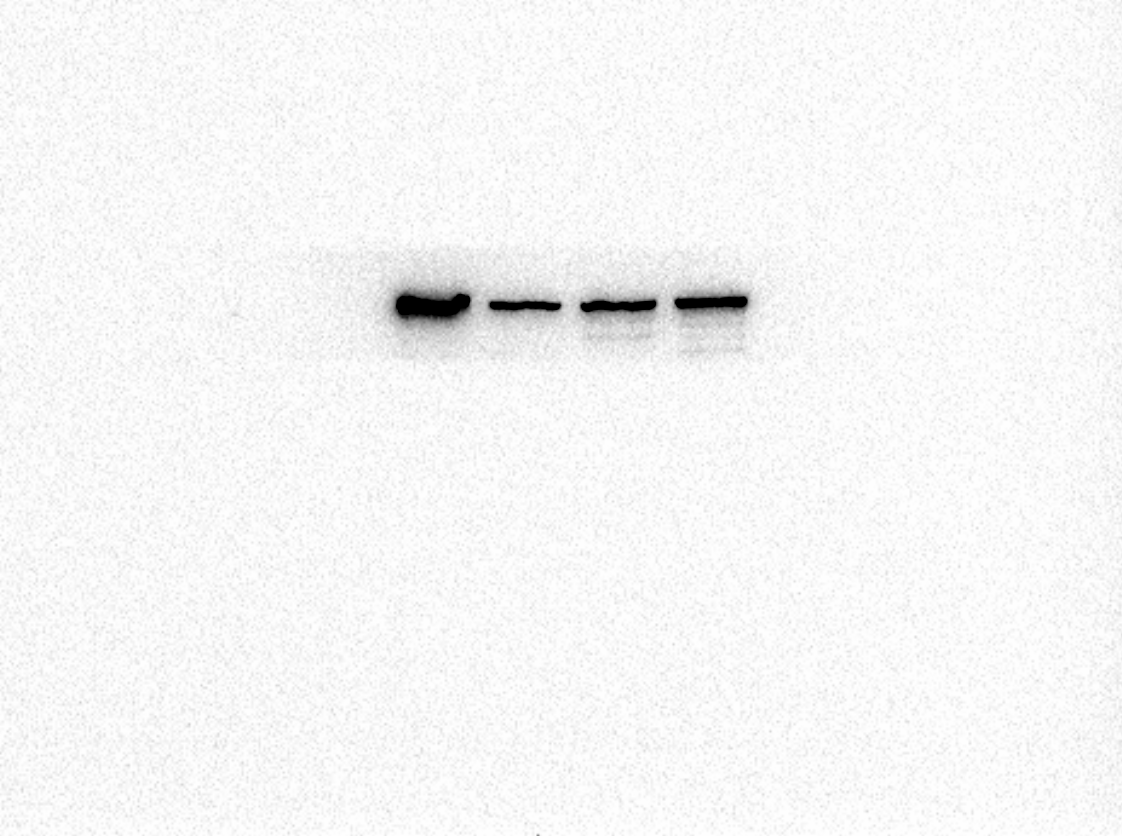

Supplement: Supplementary Figure 1 — Protein level of Eg5. Proteins were extracted from cells transfected with Eg5 si-RNA.Western blot assays were used to demonstrate the protein level of Eg5. GAPDH was used as an internal control. Data represent mean ± SD (n = 3). (***p<0.001). [file DataSheet_1.zip › supplementary results/S2/S2 Eg5.tif]

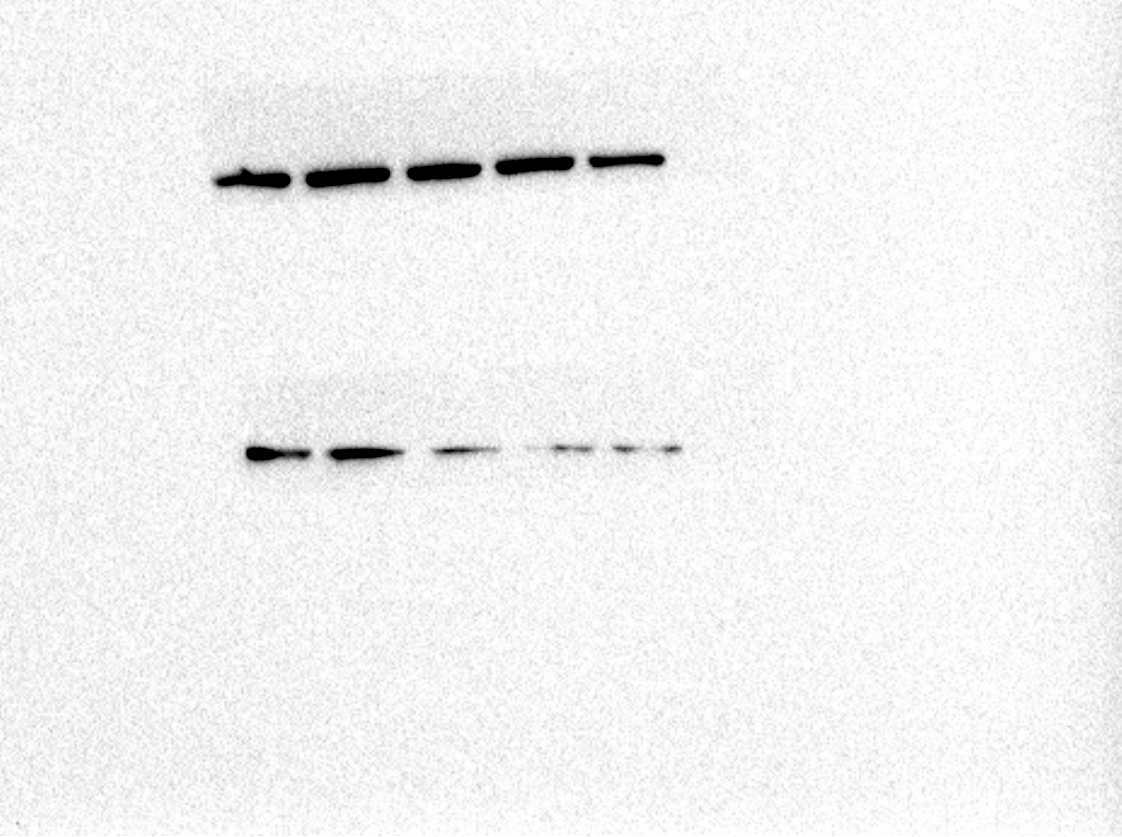

Supplement: Supplementary Figure 1 — Protein level of Eg5. Proteins were extracted from cells transfected with Eg5 si-RNA.Western blot assays were used to demonstrate the protein level of Eg5. GAPDH was used as an internal control. Data represent mean ± SD (n = 3). (***p<0.001). [file DataSheet_1.zip › supplementary results/S2/S2 GAPDH (above).tif]

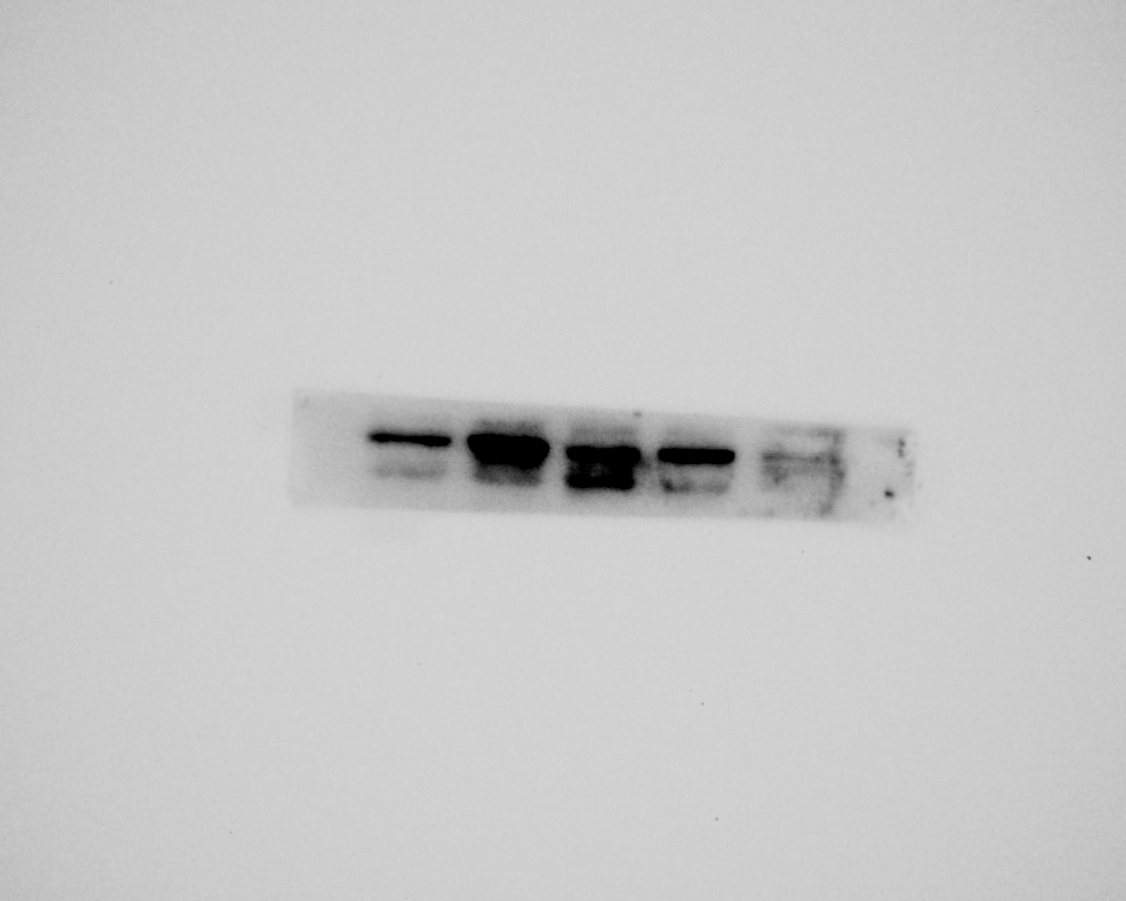

Supplement: Supplementary Figure 1 — Protein level of Eg5. Proteins were extracted from cells transfected with Eg5 si-RNA.Western blot assays were used to demonstrate the protein level of Eg5. GAPDH was used as an internal control. Data represent mean ± SD (n = 3). (***p<0.001). [file DataSheet_1.zip › supplementary results/S2/S2 Smurf2.tif]

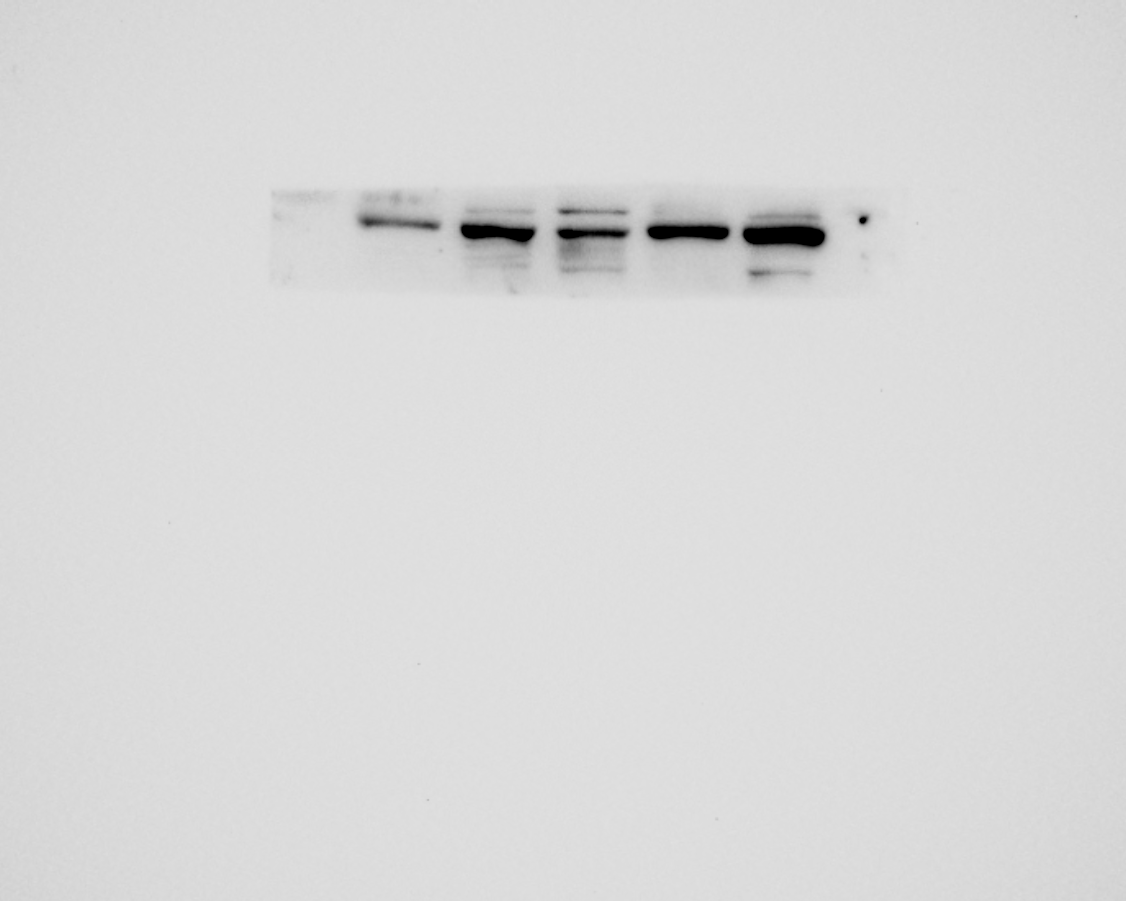

Supplement: Supplementary Figure 1 — Protein level of Eg5. Proteins were extracted from cells transfected with Eg5 si-RNA.Western blot assays were used to demonstrate the protein level of Eg5. GAPDH was used as an internal control. Data represent mean ± SD (n = 3). (***p<0.001). [file DataSheet_1.zip › supplementary results/S2/S2 TRAF4.tif]

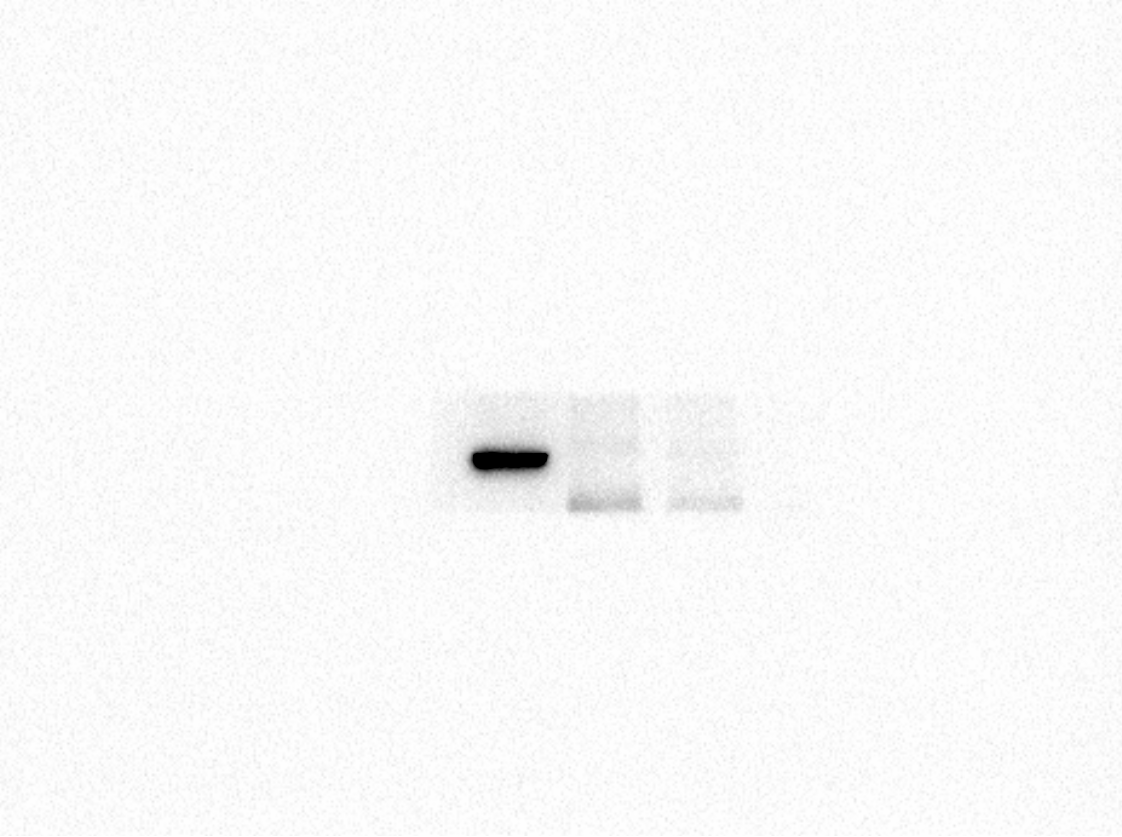

Supplement: Supplementary Figure 1 — Protein level of Eg5. Proteins were extracted from cells transfected with Eg5 si-RNA.Western blot assays were used to demonstrate the protein level of Eg5. GAPDH was used as an internal control. Data represent mean ± SD (n = 3). (***p<0.001). [file DataSheet_1.zip › supplementary results/S3/IP-Eg5 IB-actin.tif]

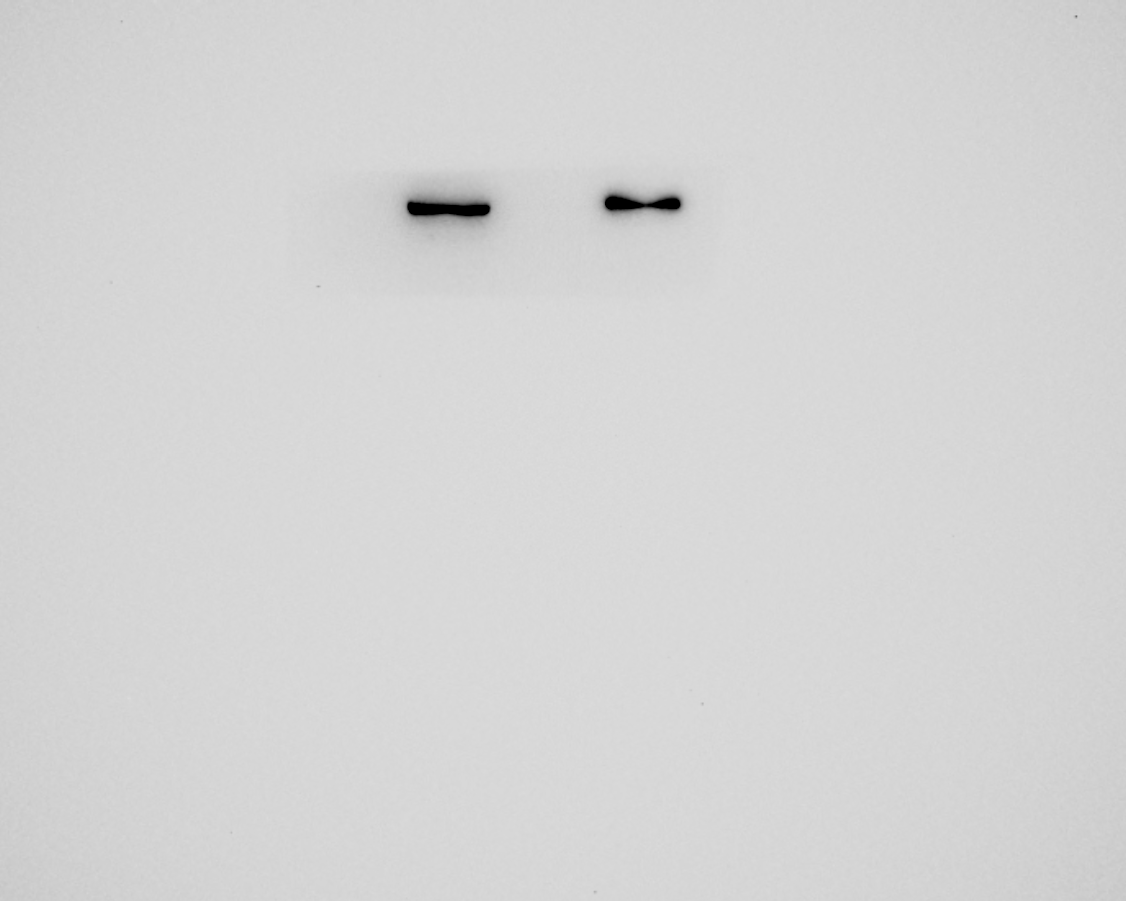

Supplement: Supplementary Figure 1 — Protein level of Eg5. Proteins were extracted from cells transfected with Eg5 si-RNA.Western blot assays were used to demonstrate the protein level of Eg5. GAPDH was used as an internal control. Data represent mean ± SD (n = 3). (***p<0.001). [file DataSheet_1.zip › supplementary results/S3/IP-Eg5 IB-Eg5.tif]

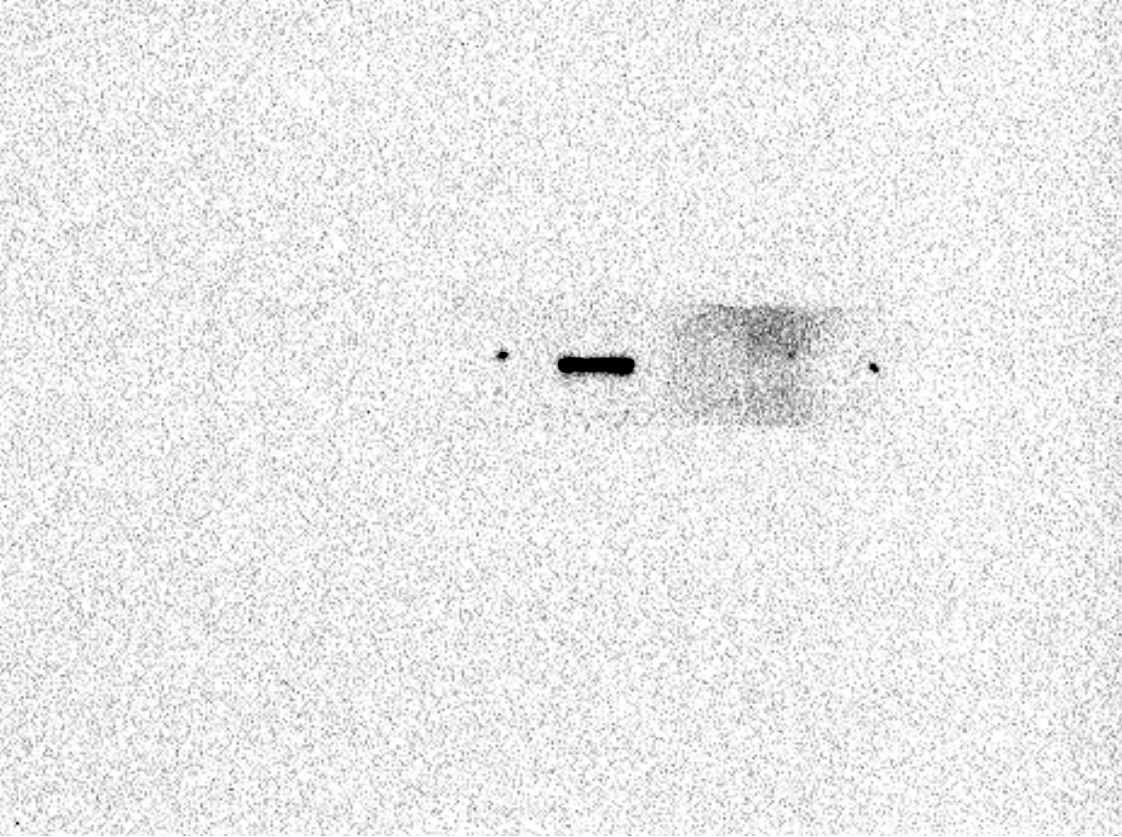

Supplement: Supplementary Figure 1 — Protein level of Eg5. Proteins were extracted from cells transfected with Eg5 si-RNA.Western blot assays were used to demonstrate the protein level of Eg5. GAPDH was used as an internal control. Data represent mean ± SD (n = 3). (***p<0.001). [file DataSheet_1.zip › supplementary results/S3/IP-Smurf2 IB-actin.tif]

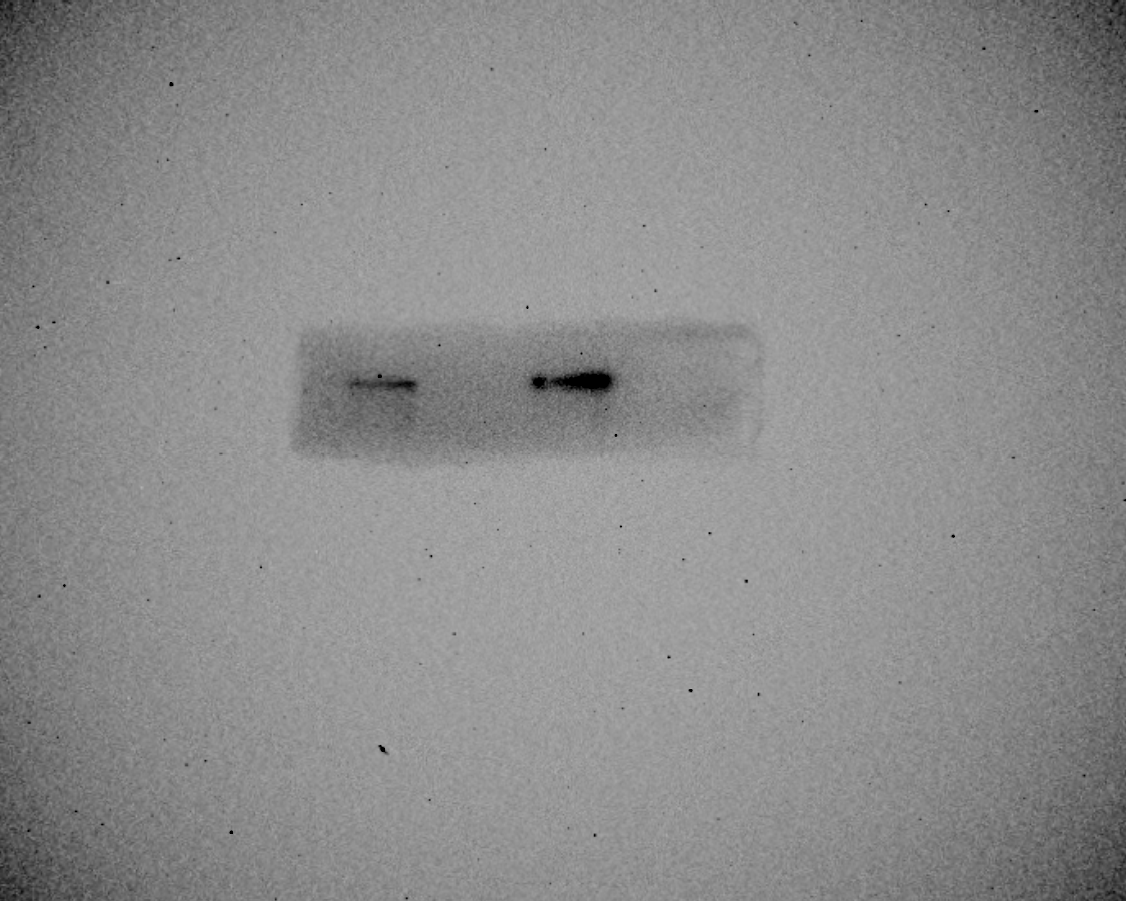

Supplement: Supplementary Figure 1 — Protein level of Eg5. Proteins were extracted from cells transfected with Eg5 si-RNA.Western blot assays were used to demonstrate the protein level of Eg5. GAPDH was used as an internal control. Data represent mean ± SD (n = 3). (***p<0.001). [file DataSheet_1.zip › supplementary results/S3/IP-Smurf2 IB-Smurf2.tif]

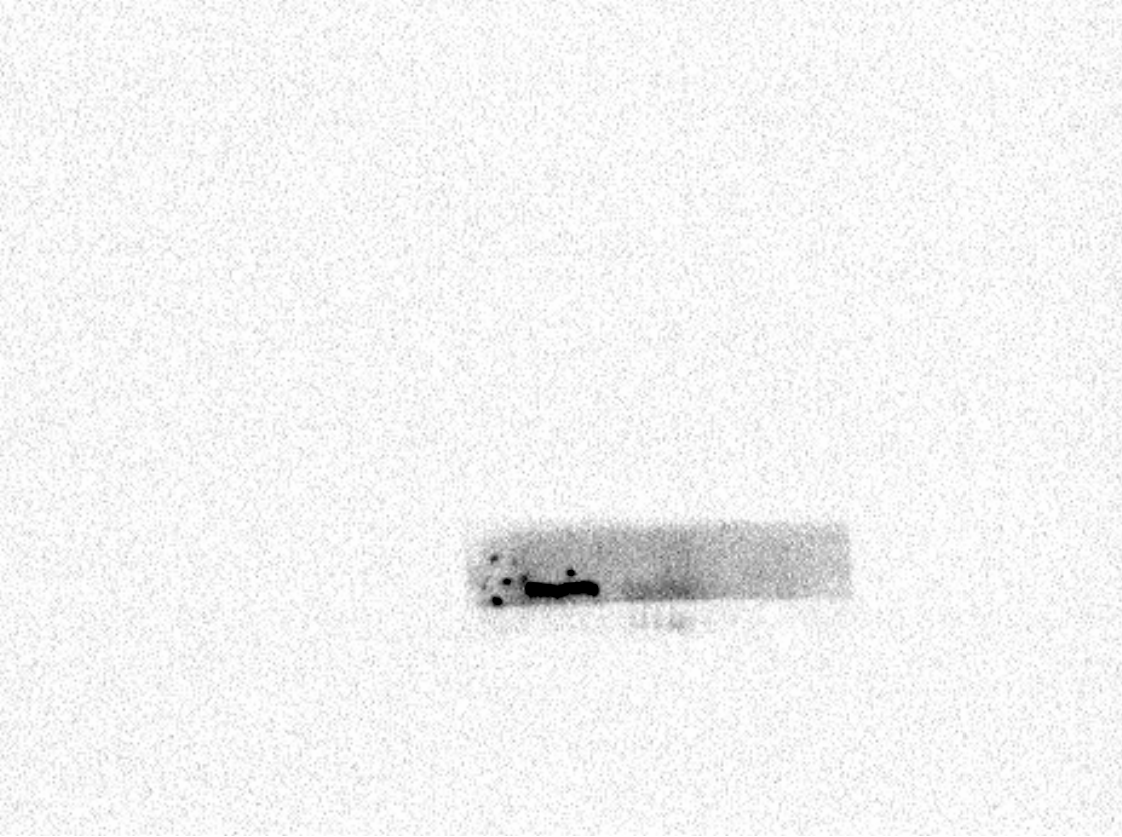

Supplement: Supplementary Figure 1 — Protein level of Eg5. Proteins were extracted from cells transfected with Eg5 si-RNA.Western blot assays were used to demonstrate the protein level of Eg5. GAPDH was used as an internal control. Data represent mean ± SD (n = 3). (***p<0.001). [file DataSheet_1.zip › supplementary results/S3/IP-TRAF4 IB-actin.tif]

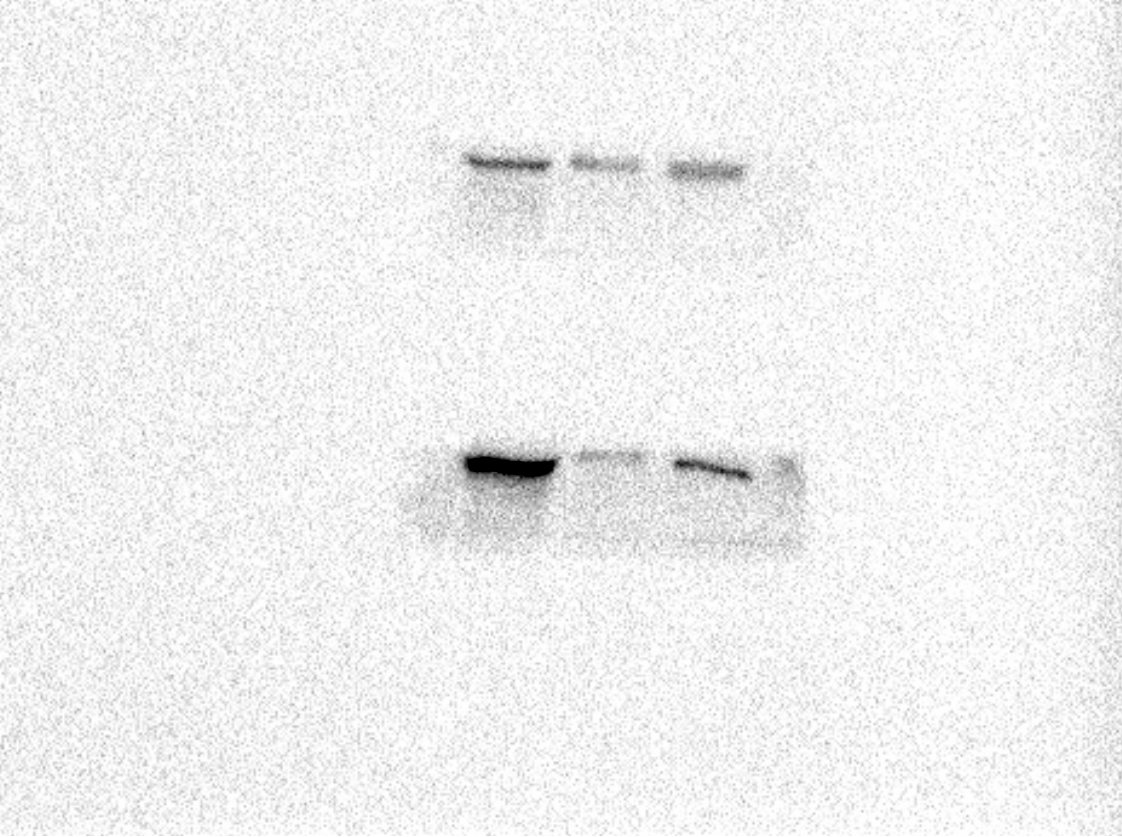

Supplement: Supplementary Figure 1 — Protein level of Eg5. Proteins were extracted from cells transfected with Eg5 si-RNA.Western blot assays were used to demonstrate the protein level of Eg5. GAPDH was used as an internal control. Data represent mean ± SD (n = 3). (***p<0.001). [file DataSheet_1.zip › supplementary results/S3/IP-TRAF4 IB-TRAF4 (bottom).tif]

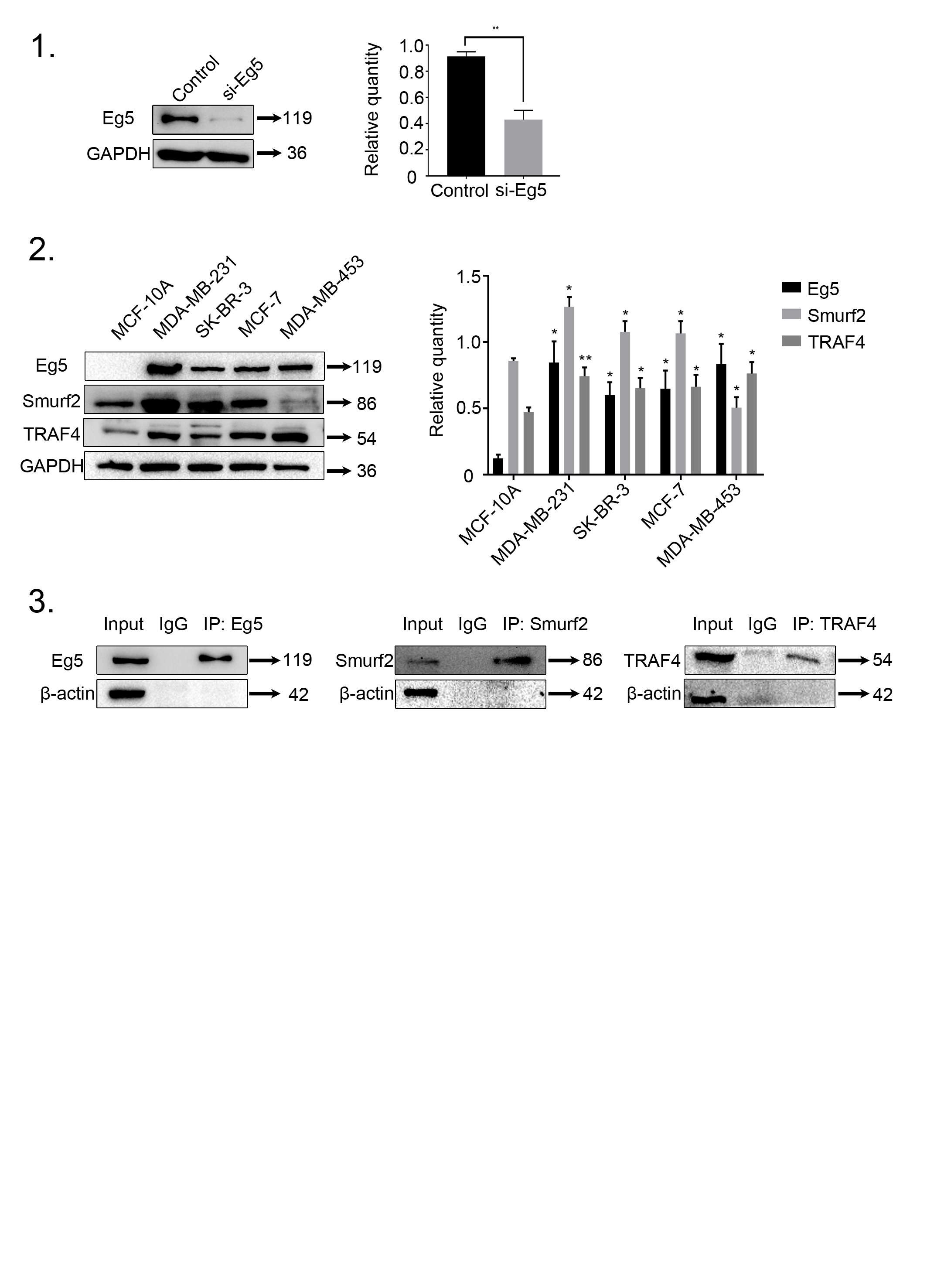

Supplement: Supplementary file 3 [file Image_1.tif]
